# Home-made low-cost dosemeter for photon dose measurements in radiobiological experiments and for education in the field of radiation sciences

**DOI:** 10.1007/s00411-024-01076-1

**Published:** 2024-06-07

**Authors:** August Blomgren, Adrianna Tartas, Prabodha Kumar Meher, Samuel Silverstein, Andrzej Wojcik, Beata Brzozowska

**Affiliations:** 1 Department of Physics, Stockholm University, Roslagstullsbacken 21, 114 21 Stockholm, Sweden; 2 Biomedical Physics Division, Faculty of Physics, Institute of Experimental Physics, University of Warsaw, Pasteura Street 5, 02-093 Warsaw, Poland; 3 Department of Molecular Biosciences, The Wenner-Gren Institute, Stockholm University, Svante Arrhenius väg 20C, 114 18 Stockholm, Sweden; 4 Institute of Biology, Jan Kochanowski University, Uniwersytecka Street 7, 25-406 Kielce, Poland

**Keywords:** Dosimetry, Radiobiology, Photon radiation, Silicon detector, Radiochromic films, Ionization chamber

## Abstract

Reliable dosimetry systems are crucial for radiobiological experiments either to quantify the biological consequences of ionizing radiation or to reproduce results by other laboratories. Also, they are essential for didactic purposes in the field of radiation research. Professional dosemeters are expensive and difficult to use in exposure facilities with closed exposure chambers. Consequently, a simple, inexpensive, battery-driven dosemeter was developed that can be easily built using readily available components. Measurements were performed to validate its readout with photons of different energy and dose rate and to demonstrate the applicability of the dosemeter. It turned out that the accuracy of the dose measurements using the developed dosemeter was better than 10%, which is satisfactory for radiobiological experiments. It is concluded that this dosemeter can be used both for determining the dose rates of an exposure facility and for educational purposes.

**Supplementary Information:**

The online version contains supplementary material available at 10.1007/s00411-024-01076-1.

## Introduction

Ionising radiation is used in biological experiments for various purposes. In the field of radiation protection research, the relationship between a radiation dose and the magnitude of the studied biological effect is important to understand the mechanism of action and infer potential health risks. In the broader field of toxicology, radiation is often used as a source of damage induced in cellular organelles to study the cellular response. The advantage of radiation over chemical toxic agents is that the level of damage and the timing of its induction can be controlled with great accuracy. Whatever the purpose, the dose and its possible spatial variation delivered to any biological sample should be determined and described.

Several authors have pointed out that proper dosimetric information is often missing in radiobiology publications (Desrosiers et al. 2013; Pedersen et al. 2016; Sefl et al. 2018; DeWerd and Kunugi 2021; Wojcik et al. 2024). Reasons include lack of access to appropriate dosimetric equipment and insufficient knowledge in the field of dosimetry. The consequence is that published results are often difficult to be reproduced by other laboratories, and to be compared with those using other biological materials (Pedersen et al. 2016; Claridge Mackonis et al. 2018).

Reliable dosimetry systems are active or passive (Kron et al. 2016). Active dosemeters provide a direct display of the dose rate or accumulated dose, but apart from battery-driven devices that show the personal dose equivalent, they typically require a cable connecting to the electrical outlet and, in case of some devices, a cable connecting a semiconductor or ionization chamber detector. This makes such systems unsuitable for dosimetric measurements within irradiators with closed exposure chambers, such as the Gammacell® 40 Exactor (Best Theratronics Ltd., Ottawa, Canada). Such irradiators have ports for guiding cables into the radiation chambers, but they are designed for servicing purposes and are, therefore, too narrow for an ionisation chamber or a cable connector. The dose rate inside the exposure chambers is usually in the order of 1 Gy/min, a value that is optimal for radiobiology experiments typically requiring doses in the range of a few Gy. In such experiments, short exposure times are important because cells cultured under in vitro conditions should not be kept outside an incubator for times longer than a few minutes. These dose rates and doses used in those experiments are, however, beyond the range of battery-driven personal dosemeters such as RadEye (Thermo Fisher Scientific Inc). Passive dosemeters such as radiographic films [for example Gafchromic (Papaconstadopoulos et al. 2014)], thermoluminescent detectors, alanine detectors, or Fricke dosemeters (deAlmeida et al. 2014) can be placed in closed exposure chambers, but the interpretation of the signal requires dedicated equipment and know-how that is often not available in radiation biology laboratories.

Furthermore, while the curriculum in education of medical physicists contains exhaustive theoretical and practical training in dosimetry, that of radiation biologists does not. Consequently, many radiation biologists have a limited understanding of dosimetry and, consequently, for their experiments they rely on historical dosimetric information of exposure devices or dose rates given by the producer of purchased equipment. Theoretical knowledge can be gained through literature studies, but practical experience requires access to dosemeters, and appropriate equipment is often not available in radiation biology laboratories. Moreover, as described above, most active dosemeters are not suitable for dosimetric measurements on irradiators with closed exposure chambers.

With this in mind a simple, inexpensive, battery-driven dosemeter was developed that can easily be built using widely available components. Being inexpensive, the dosemeter has limitations with respect to radiation energy and sensitivity. While it is not intended for purposes of calibrating radiation sources, it is well suited for determining the homogeneity of a radiation field inside an irradiation chamber and for educational purposes. In this publication the dosemeter design is described and results of validation studies with photon sources of different energy and dose rate are presented.

## Materials and methods

### Dosemeter design

**Fig. 1:**
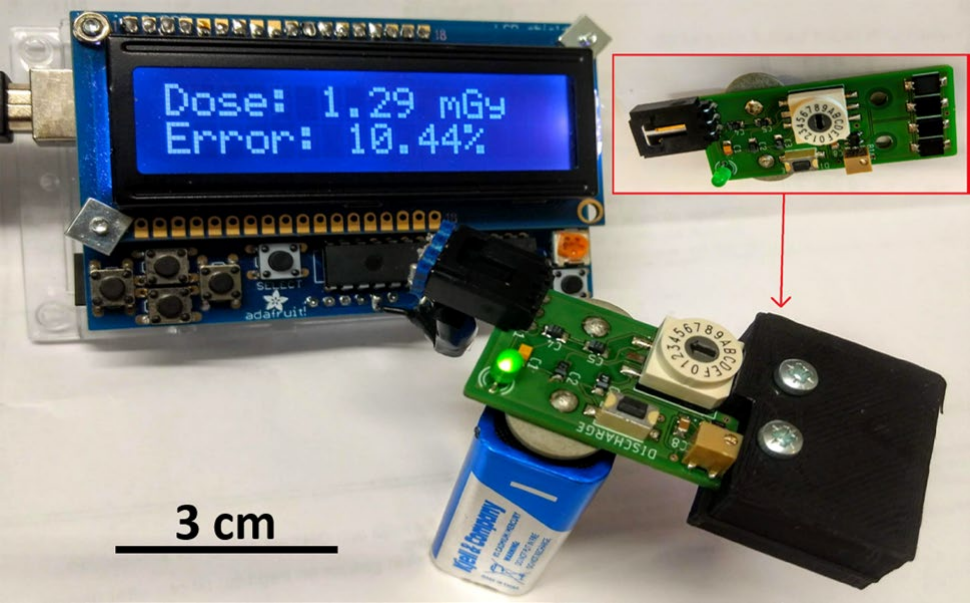
A fully assembled dosemeter mounted with the build-up cap (black part of the dosemeter, thickness: 5 mm) mounted, and a readout device that displays the measured dose together with the level of uncertainty. The uncovered dosemeter with four capacitors located to the left of the rotary switch is shown in the red frame. Just below the rotary switch to the right are the amplifier and the trimpot used to supply the bias voltage. The discharge button, which should be pressed before each measurement, is just to the left of the trimpot. A green LED on the bottom left indicates that the dosemeter is switched on

Dosemeter design, technical details and helpful user information are presented in the Electronic Supplement (Appendix A). In short, the design uses four inexpensive, high-speed and highly sensitive silicon photodiodes (Vishay Intertechnology, Inc., model VBPW34FAS) connected to a single-stage integrating amplifier (Fig. 1). The photodiodes are covered with a 3D-printed build-up cap of solid acrylonitrile butadiene styrene (ABS) plastic, which has a density close to that of water (1.0–1.05 g/cm^3^). The dosemeter board is 20 mm wide and 48 mm long, and is powered by a 9 V battery. A rotary switch connected to four integrating capacitors allows the amplifier gain to be set to an appropriate value for the expected dose range. Immediately after exposure, the dosemeter is connected to an Arduino Uno (Arduino, https://www.arduino.cc) microcontroller board to read and display the absorbed dose. This approach avoids ionizing radiation damage to the Arduino, and allows the dosemeter to be as compact as possible so that it can fit even inside small exposure chambers and be used to map dose inhomogeneities in a radiation field.

The integrated charge from any radiation exposure depends on the photon energy and the dosemeter capacitance, resulting in different ranges of dose measurements. The dose range is selected by setting the rotary switch to a given position according to the dosemeter manual (see Electronic Supplement, Appendix A). A menu on the reading device allows the user to select the source and integrator capacitance (rotary switch position) used during irradiation and includes proposed adjustments for predefined radiation sources such as ^137^Cs, ^60^Co, and an X-ray tube. There is also an option to manually enter the photon energy emitted from a radiation source. When both the source and capacitance were entered, the reading device calculates the dose according to Eq. 1:

1 $$D=\frac{\overline{U}C }{R(E){C}_{0}}$$

where $\overline{U }$ is the measured average voltage over the capacitor, $C$ is the capacitance used during the measurement, $R(E)$ is the dosemeter response to radiation depending on energy (voltage increase per unit dose for a given radiation field) and ${C}_{0}$ is the capacitance used for $R(E)$ measurement.

The uncertainty associated with the measured dose is calculated by applying the standard formula of error propagation to Eq. 1:

2 $$u_{D} = \sqrt {\left( {u_{{\overline{U}}} \frac{dD}{{d\overline{U}}}} \right)^{2} + \left( {u_{C} \frac{dD}{{dC}}} \right)^{2} + \left( {u_{R} \frac{dD}{{dR}}} \right)^{2} + \left( {u_{{C_{0} }} \frac{dD}{{dC_{0} }}} \right)^{2} } .$$

To be able to calculate *u*_D_, all sources of uncertainty had to be determined experimentally, except for the uncertainty in the capacitance, which was provided in the capacitor data sheet.

Ten identical dosemeters were constructed and used in the present study.

### Radiation sources

Three radiation sources were used in the validation study of the dosemeters: two irradiators with ^137^Cs sources, a ^60^Co source, and an X-ray source.

#### ^137^Cs sources

The irradiators including ^137^Cs sources are installed at Stockholm University, Sweden. The first irradiator used was a Scanditronix IC900 irradiator (Scanditronix AB, Uppsala, Sweden). It is a closed-chamber exposure facility with two opposing 16.65 TBq ^137^Cs sources and a table to position samples between them. ^137^Cs is an emitter of gamma rays with a peak energy of 662 keV and a physical half-life of 30 years. At the time of the measurements, the dose rate on the sample table was 0.372 Gy/min.

The second irradiator was a Gammacell 40 Exactor (AECL, Canada), which is a research irradiator. The uniformity of the dose inside the exposure chamber is ensured by two sources of ^137^Cs, each having an activity of 55.5 TBq. At the time of the measurements, two dose rates inside the exposure chamber were used: 0.356 Gy/min and 0.79 Gy/min. The exposure chambers of both sources must be tightly shut when in operation and, consequently, dose monitoring with an ionization chamber is not possible. All sources have been calibrated by Fricke dosimetry in 2018 (Olofsson et al. 2020).

#### ^60^Co source

The ^60^Co source was a Theratron Elite 80 ^60^Co source (MDS Nordion, Canada) that is installed at the Karolinska University Hospital, Stockholm, Sweden. This source is a former therapy source for external beam radiotherapy currently used for experimental purposes. It is operated inside a dedicated bunker where the angle of the gantry and the distance between the source and the sample can be freely adjusted, confined by the size of the bunker. The distance between the source and the sample was set at 80 cm and the field size was 15 × 15 cm. ^60^Co emits gamma rays with energies of 1173 keV and 1332 keV and can be considered as a monoenergetic 1250 keV source. The physical half-life of ^60^Co is 5.3 years. At the time of the measurements, the dose rate at the sample position was 0.070 Gy/min, determined with a Unidos E dosemeter equipped with a TM30010 ionization chamber, PTW, Freiburg, Germany.

#### X-ray source

The X-ray source was included in a YXLON SMART 200 machine (Yxlon International, Hamburg, Germany), which is installed at Stockholm University, Sweden, and described in detail in (Staaf et al. 2012) and (Brehwens et al. 2012). Measurements were performed for a tube voltage of 190 kV and a tube current of 4.0 mA with a 5 mm Al filter, leading to photon emissions with a maximum energy of 190 keV and a peak energy of 80 keV (Brehwens et al. 2012). The distance between the X-ray source and the sample was 40 cm. The dose rate was 0.068 Gy/min as determined with a Unidos E dosemeter equipped with a TM30010 ionization chamber, PTW, Freiburg, Germany.

### Validation of dose measurements

Doses due to exposures with the ^137^Cs and X-ray sources measured with one dosemeter were validated by dosimetric measurements using a Gafchromic film. The Gafchromic film was calibrated for X-rays using an ionization chamber dosemeter. Note that the dose response of radiochromic films is independent of the energy or dose rate (see Electronic Supplement, Appendix B).

#### Radiochromic film calibration with ionisation chamber

In order to calibrate the Gafchromic film EBT3 (Ashland Inc., Bridgewater, NJ), the dose rate of the X-ray source was determined using a certified cylindrical ionization chamber (Unidos E equipped with a TM30010 ionization chamber, PTW, Freiburg, Germany) with a nominal sensitive volume of 0.6 cm^3^. The calibration was carried out at Stockholm University and the X-ray source was chosen because, unlike the ^137^Cs source, it is not operating in a closed chamber, so dose measurements with an active dosemeter were possible. The dose response curve for the EBT3 Gafchromic films was determined by exposing 2.5 × 2 cm film strips at times corresponding to doses between 0 and 2 Gy and scanning the strips 24 h after the end of irradiation. A flatbed Epson Expression 12000XL scanner (Epson Seiko Epson Corp., Nagano, Japan) was used to read the films (Cho et al. 2020). RGB TIF images were analyzed using the ImageJ software (version 1.53i, Java 1.8.0_172, Wayen Rasband, National Institutes of Health, USA; website: http://rsb.info.nih.gov/ij/download.html). Calculations of pixel intensity were done based on the red channel only, which is known to possess the highest dose sensitivity (Stevens et al. 1996; Ohuchi 2007). The pixel intensity was transformed into net optical density (*netOD*) according to Eq. 3:

3 $$netOD={log}_{10}\left(\frac{I-{I}_{bcg}}{{I}_{0}-{I}_{bcg}}\right)$$

where *I* and *I*_*0*_ correspond to readings for irradiated and nonirradiated Gafchromic films and *I*_*bcg*_ is the background reading (signal of a blank scan) without the Gafchromic film (Papaconstadopoulos et al. 2014). The *netOD* dose response was fitted to a linear function according to Eq. 4:

4 $$netOD=a{D}_{IC}+b$$

where *D*_*IC*_ is the dose determined from measurements with the ionization chamber, and $a$ and $b$ are the fitting parameters. The calibration curve was used to validate both X-ray and gamma radiation measurements that had been performed with the semiconductor dosemeters. This approach is justified by the fact that the sensitivity of radiochromic films to gamma radiation is independent of the photon energy (Rink et al. 2007) and its dose rate (see Electronic Supplement, Appendix B).

### Comparison of X-ray dose measurements using a dosemeter and Gafchromic films

The doses from the X-ray source (used for calibration) and the ^137^Cs source were measured with EBT3 Gafchromic films and a randomly selected dosemeter. For the X-ray source, measurements were performed for four irradiation times: 221, 441, 882, and 1,765 s corresponding, respectively, to 0.25, 0.5, 1.0 and 2.0 Gy. For the ^137^Cs source, measurements were also performed for four irradiation times: 42, 84, 169 and 337 s corresponding to 0.25, 0.5, 1.0 and 2.0 Gy, respectively. Performing measurements for such a wide range of doses required selecting the right position of the rotary switch. The doses recorded by the dosemeter were read immediately after the irradiation was complete. For X-rays, measurements were made several times. First, a series of measurements was made for different doses, at a dose rate of 0.068 Gy/min, and then for a dose of 1 Gy, but for different dose rates. Different dose rates were obtained by changing the distance between the source and the dosemeter. This was done to test the detector's response to different X-ray dose rates.

### Measurement reproducibility and charge leakage

To test the reproducibility of the dose measurements, a series of measurements were performed with two instruments exposed to ^60^Co, ^137^Cs and X-rays.

#### Reproducibility of measurement

^137^Cs, ^60^Co and X-ray sources were used to verify the reproducibility of a measurement. Two randomly chosen dosemeters were repeatedly exposed 14 times to the ^137^Cs source, six times to the ^60^Co source and 10 times to the X-ray source. The dose and exposure settings were kept constant for each exposure with one source.

#### Charge leakage

Charge leakage (drift) was determined with all dosemeters at a background radiation field with a dose rate of 0.2 µSv/h (personal dose equivalent per hour) over a time of 150 min. It was found to be stable at 1.57 mGy/min, with a standard deviation of 0.11 mGy/min.

### Statistical analyses

For radiochromic film calibration with an ionization chamber a linear function in the form of $y=ax + b$ was fitted to measured data points, where $y$ represents optical density, $x$ represents the dose measured with the ionisation chamber, $a$ is the slope of the line, and $b$ is the y-intercept. Goodness-of-fit of the *netOD* values to the X-ray and gamma dose was estimated by calculating the coefficient of determination *R*^2^. Uncertainties were calculated using the error propagation method. The doses estimated by the semiconductor dosemeters and Gafchromic films were compared by applying a two-sided, paired Student´s t-test. All calculations were performed with Python software (https://www.python.org).

### Dose homogeneity measurements inside irradiation chambers

**Fig. 2:**
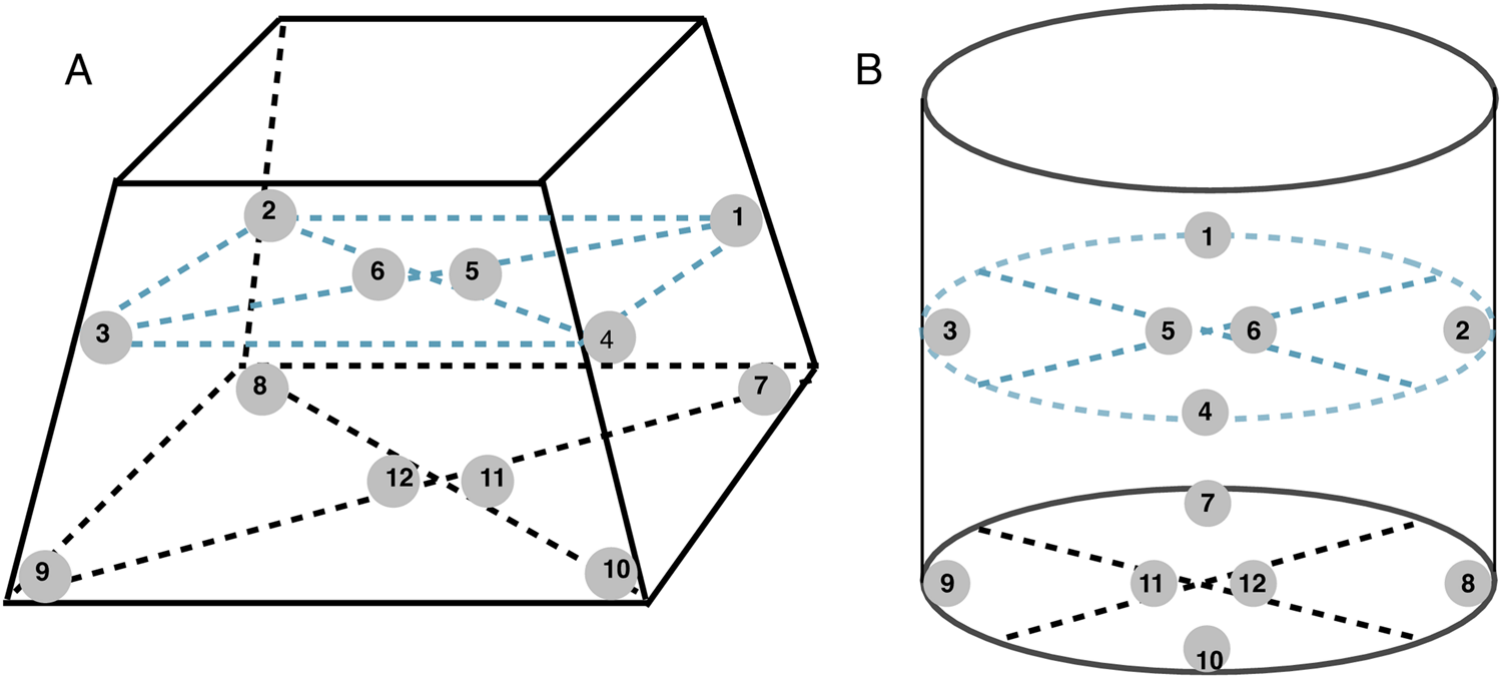
Positions of dose measurements in the Scanditronix (**A**) and Gammacell (**B**) irradiation chambers

Spatial dose distribution measurements were performed with randomly selected semiconductor dosemeters placed in two gamma irradiation chambers: Scanditronix and Gammacell. As shown in Fig. 2 dosemeters were placed in 12 positions in the irradiation chamber: six in the plane at the bottom of the chamber and six in the plane located at half of the chamber height. The mean and variation of doses were calculated for both chambers. The reference dose was equal to 2 Gy.

## Results

### Repeatability of the measurements

**Fig. 3:**
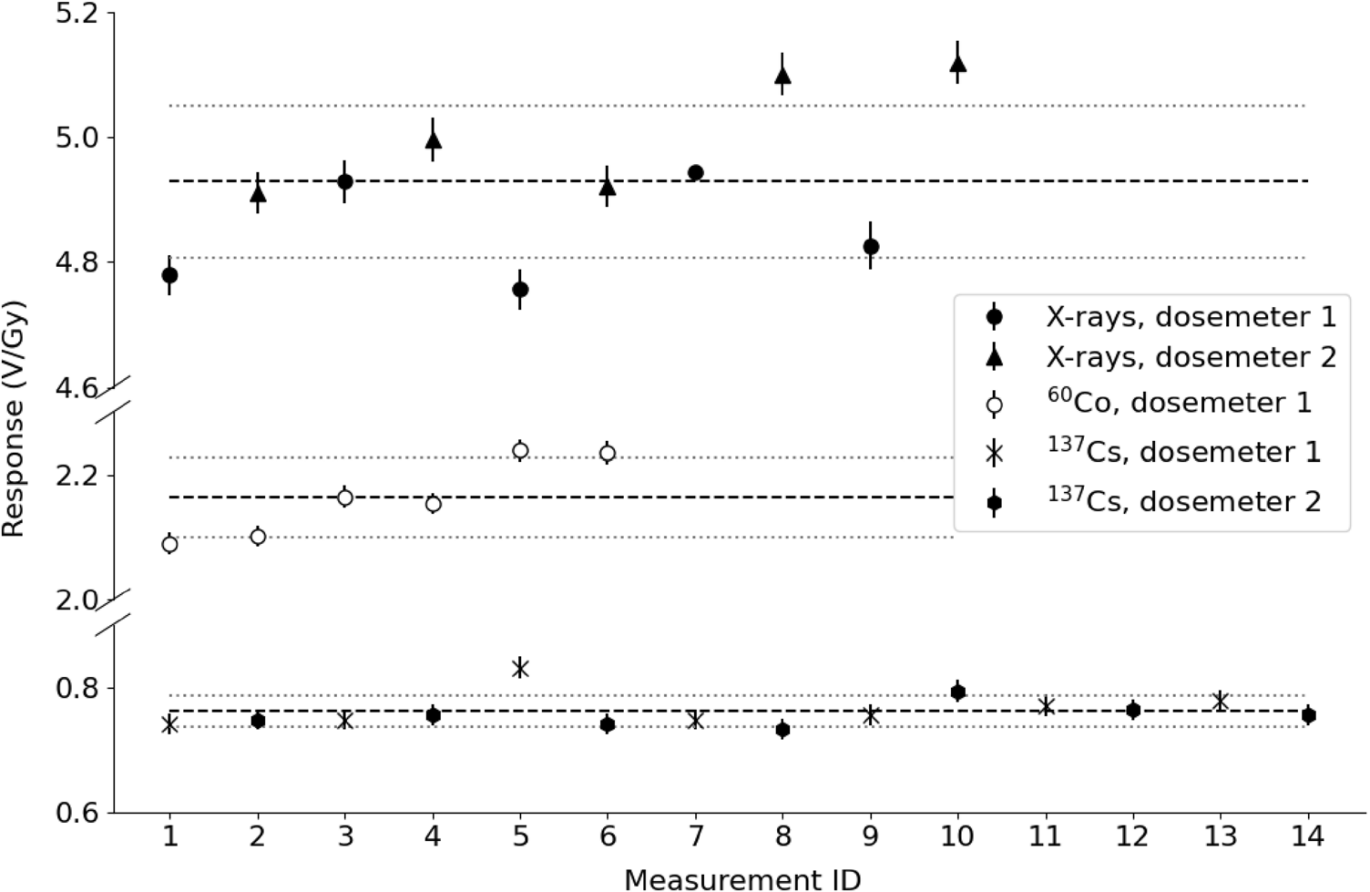
Dosemeter response when exposed to X-rays and gamma radiation. Solid circles and triangles represent 10 X-ray measurements using two dosemeters; open points show six measurements for the ^60^Co source using one of these dosemeters; x-marks and solid hexagons represent 14 measurements for the ^137^Cs source using two dosemeters. The lines denote the mean of the measurements (dashed) and the standard deviation of the mean (dotted). Error bars represent uncertainties given by the dose reading device (Eq. 2)

Measurements were performed with one (for ^60^Co) or two (for X-rays and ^137^Cs) randomly selected dosemeters to test the repeatability of response to 1 Gy of X-rays, 1 Gy of ^60^Co gamma radiation and 1 Gy of ^137^Cs gamma radiation. The individual measurement results are shown in Fig. 3, expressed as V/Gy. The response was equal to (4.93 ± 0.12) V/Gy for X-rays, (2.165 ± 0.063) V/Gy for ^60^Co and (0.76 ± 0.03) V/Gy for ^137^Cs gamma radiation. Regardless of the type of radiation, the differences between the dosemeter readouts were within 3%.

### Validation of dose measurements

#### Comparison of dose measurements using semiconductor dosemeters and Gafchromic films

**Fig. 4:**
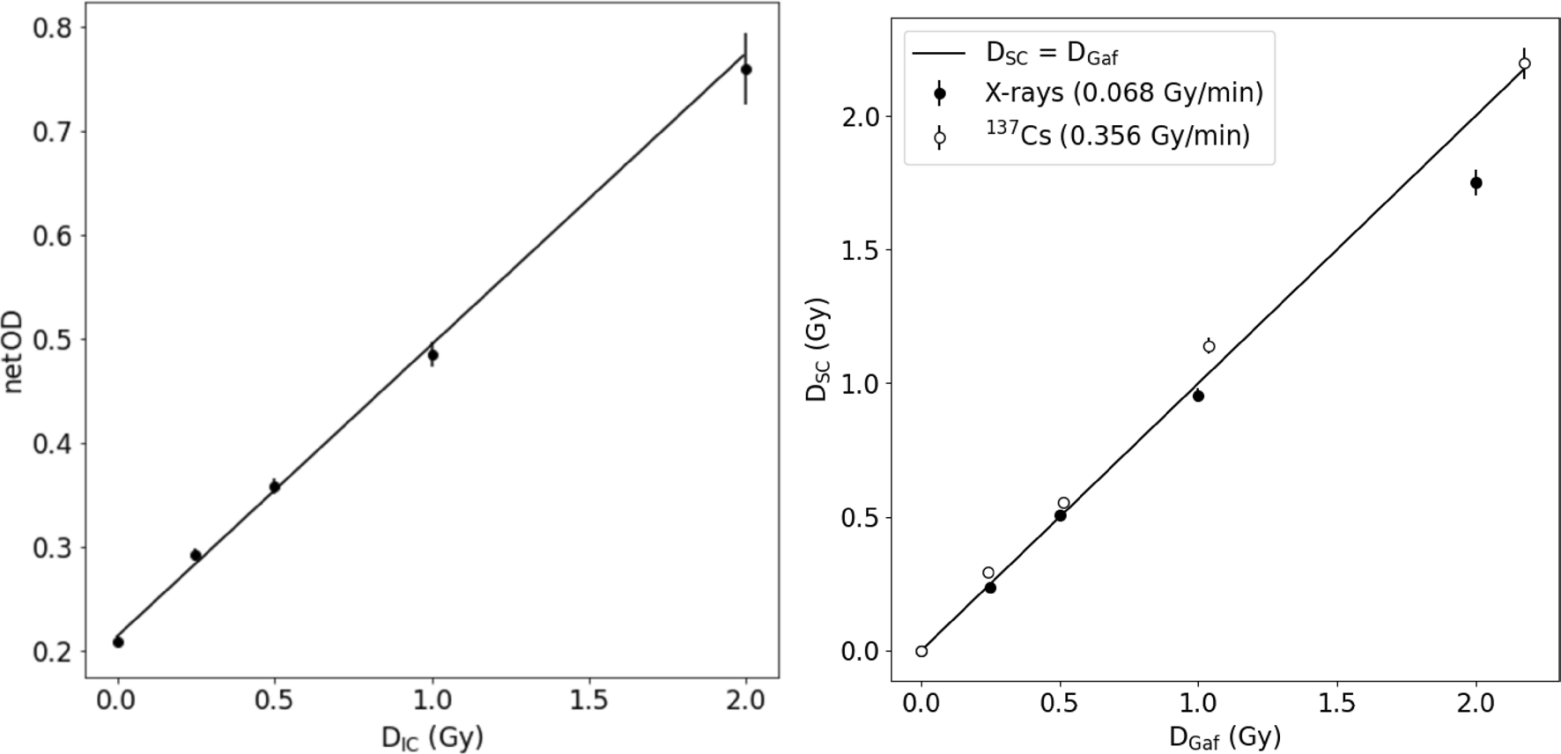
Dose measurements performed with a semiconductor dosemeter (D_SC_) as a function of the reference dose (D_Gaf_) (right panel); D_Gaf_ was obtained using the film calibration curve shown on the left panel. *D*_*IC*_ dose measured with an ionization chamber; *netOD* net optical density. Error bars represent standard deviations

Based on one radiochromic film calibration curve (Fig. 4, left panel), reference dose values were determined for all photon sources (Rink et al. 2007). To obtain this calibration curve, optical densities of the Gafchromic film were determined after exposure to various X-ray doses, which were in turn measured with an ionization chamber. Data points were fitted using Eq. 4. In Fig. 4, error bars represent uncertainties calculated by the error propagation method. The resulting fit parameters were $a$ = 0.2132 ± 0.0046 and $b$ = 0.281 ± 0.012. The coefficient of determination R^2^ was 0.99 demonstrating a very good fit of the *netOD* values to the X-ray dose.

X-ray doses estimated by a randomly selected dosemeter (D_SC_) were compared with doses estimated using Gafchromic films (D_Gaf_). The results are presented in Table 1. As can be seen, for X-rays the semiconductor doses were systematically lower by (5.0 ± 5.3) percent as compared to the film doses. These differences were significant (p-values are < 0.05) for 882 s and 1765 s of irradiation time. In contrast, for ^137^Cs gamma radiation the semiconductor doses were systematically higher by (8.0 ± 4.2) percent as compared to the film doses. The difference were significant (p-values are < 0.05) for 84 s and 169 s of irradiation time.

**Table 1:** X-ray and ^137^Cs gamma radiation doses measured with Gafchromic films (D_Gaf_) and semiconductor dosemeters (D_SC_) for a given exposure time

Time of exposure (s)	D_Gaf_ (Gy)	D_SC_ (Gy)	Percentage deviation	p-values
X-rays				
221	0.25 ± 0.03	0.24 ± 0.01	+ 4	0.6
441	0.50 ± 0.02	0.51 ± 0.02	– 2	0.4
882	1.00 ± 0.03	0.95 ± 0.04	+ 5	0.006*
1765	2.00 ± 0.08	1.75 ± 0.08	+ 13	< 0.001*
^137^Cs gamma radiation				
42	0.28 ± 0.01	0.29 ± 0.01	– 4	0.09
84	0.51 ± 0.01	0.56 ± 0.03	– 10	< 0.001*
169	1.04 ± 0.01	1.14 ± 0.05	– 10	< 0.001*
337	2.18 ± 0.03	2.20 ± 0.10	– 0.1	0.3

P-values < 0.05 marked as *, indicate a significant difference between measured doses. The sign in front of the percent deviation shows the direction of deviation

Doses of X-ray and ^137^Cs gamma radiation measured by the semiconductor dosemeters and Gafchromic films are graphically presented in Fig. 4 (right panel). The underestimation of the X-ray dose (especially with the higher doses around 2 Gy) and the slight overestimation of ^137^Cs gamma radiation are visible. The main difference between these two radiation sources, except for radiation quality, is the time the dose is delivered. For this reason, it was useful to check whether the different dose rates might have affected the dosemeter readouts.

#### Impact of dose rate

**Fig. 5:**
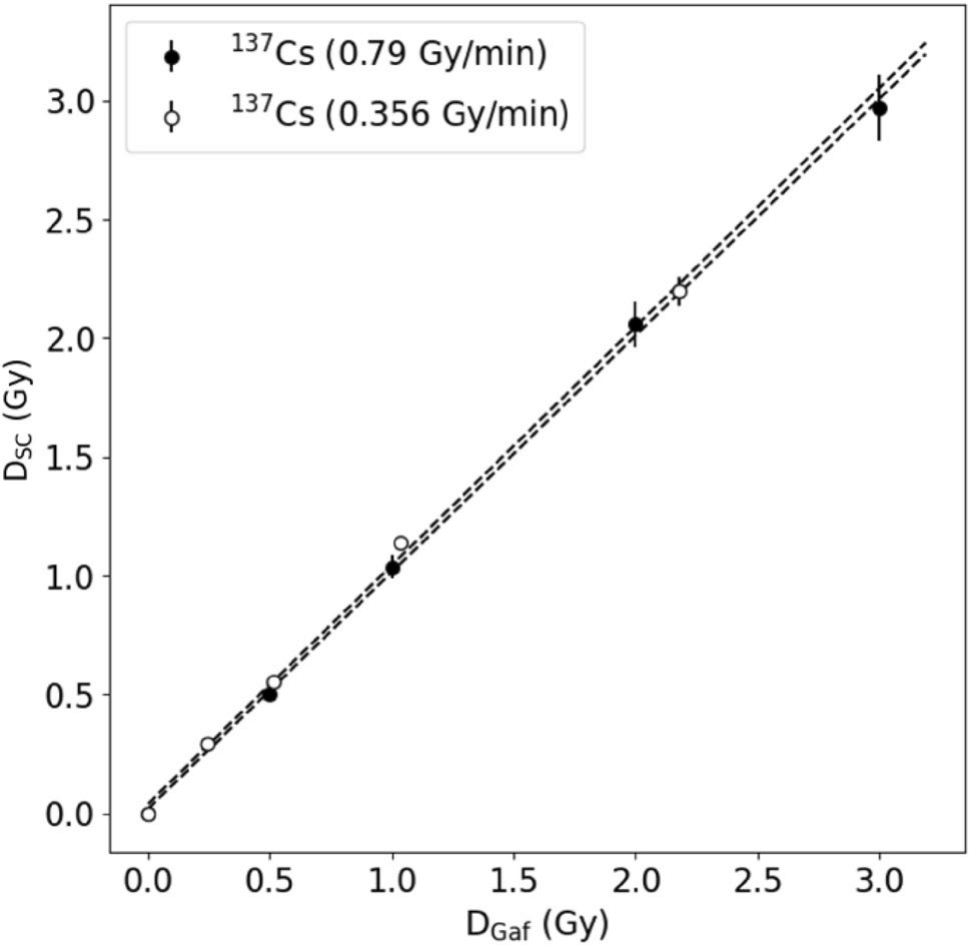
Dose measurements performed with a semiconductor dosemeter (D_SC_) and Gafchromic films (D_Gaf_) for gamma radiation from two ^137^Cs sources. Error bars represent standard deviations

Due to differences in the reading of the semiconductor dosemeters, measurements were made for different dose rates of a given photon radiation. In Fig. 5, the detector response to gamma radiation emitted from ^137^Cs sources of dose rates equal to 0.79 Gy/min and 0.356 Gy/min is shown as a function of dose.

**Fig. 6:**
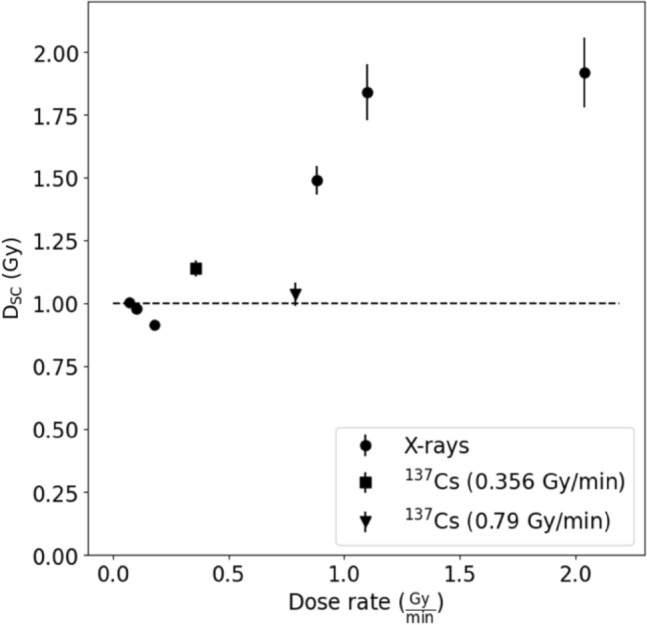
Dose measurement by a semiconductor dosemeter (D_SC_) of 1 Gy X-ray and gamma radiation performed for different dose rates. Error bars represent standard deviations

As shown in Fig. 5, for both dose rates the readings of the semiconductor dosemeter were consistent with those of the Gafchromic films, for gamma radiation. Analogically, measurements were also made for six different dose rates of a dose of 1 Gy emitted from the X-ray tube (Fig. 6).

From Fig. 6 it is evident that the dose detected by the semiconductor dosemeters for X-rays with a dose rate ≥ 1 Gy/min was about two times higher than that for gamma and low dose-rate X-ray radiation. It thus appears that the semiconductor dosemeters are about two times more sensitive to X-rays (which had an energy corresponding to 80 kVp) than to gamma radiation. The reason for the discrepancy could be the divergent radiation field present during the measurements, which is a deviation from the conditions under which the semiconductor dosemeters were calibrated.

### Spatial dose distribution in irradiation chambers

Mean doses measured in the bottom, middle and both parts of the the Scanditronix and Gammacell irradiation chambers (see also Fig. 2) are presented in Table. 2. The data shown demonstrate that the dose variation measured with the semiconductor dosemeters was below 10%. Although the doses in Scanditronix showed smaller standard errors of the mean than those measured in the Gammacell irradiator, the mean dose is higher than the reference dose of 2 Gy used in the experiment. It appears that the dose homogeneity in Gammacell is worse than that in Scanditronix, and the mean dose obtained for the Gammacell irradiator is lower than the reference dose.

**Table 2:** Mean doses measured with the semiconductor dosemeters in different parts of the gamma irradiation chambers used at Stockholm University (see Fig. 2), and corresponding standard error of the means

Irradiation chamber	Positions	Mean (Gy)	Standard error (Gy)
Scanditronix	1–6	2.16	0.10
	7–12	2.12	0.06
	1–12	2.14	0.08
Gammacell	1–6	1.81	0.12
	7–12	2.08	0.15
	1–12	1.95	0.19

## Discussion

The aim of this work was to demonstrate the design and operation of a new semiconductor dosemeter developed for controlling dose measurements in radiobiology experiments. The instrument is not meant to be used for the calibration of radiation sources, but rather for the determination of dose modifying factors such as distance from the source or shielding of an exposed sample. In biological experiments, cell samples on Petri-dishes or flasks are often stacked on top of each other and the differences in dose rate between dishes located on the top and bottom within an irradiator are often underestimated. Many radiation exposure facilities, such as the ^137^Cs sources that have been used in the present study, include two opposing radiation sources and the spatial distribution of dose rate inside the irradiation chamber is difficult to predict. An additional problem is that the chambers are tightly locked during operation and the user has no possibility to measure the dose rate with an ionisation chamber that is attached to further equipment by a cable. Ports typically included in radiation irradiators are too narrow to push through a cable connector. Consequently, the user has to rely on either passive dosemeters or battery-driven devices. Passive dosemeters based on thermoluminescence or electron paramagnetism require dedicated metrology laboratories. Battery-driven devices such as personal dosemeters reporting doses in µSv/h are too sensitive for the exposure conditions typically applied in radiobiology experiments. The semiconductor dosemeter described here is sensitive to radiation in the relevant dose and dose rate range, can easily be constructed, and is inexpensive enough to be produced in several copies. This allows simultaneous measurements with several devices placed in an irradiation chamber during one irradiation session. Apart from their application in experimental work, the dosemeters can also be used for didactic purposes to demonstrate such dose modulating factors as distance from the source or shielding. Based on didactic work the authors know that many radiation biologists have only theoretical knowledge of dosimetry and of the impact of dose-modifying factors.

The dosimetric measurements performed with the newly developed semiconductor dosemeters were validated using two types of alternative dosemeters: an ionization chamber and Gafchromic films, commonly used in clinics or dosimetry laboratories. Three sources that generate gamma radiation (^137^Cs and ^60^Co) or X-rays were used for testing the semiconductor dosemeters. The repeatability of measurements using the semiconductor dosemeters was around 3%, independently of the energy of gamma radiation. Additionally, the dose differences actually measured for a given dose for different types of radiation (X-rays and gamma radiation) was also about 3%. It was noted that the energy response of the developed semiconductor dosemeters is lower for gamma radiation as compared to the lower-energy X-ray radiation fields (such as 40–100 keV). With respect to the dose rate, the dosimeter performed well at dose rates between 0.07 Gy/min (gamma radiation and X-rays) and 0.8 Gy/min (gamma radiation). Dose rates outside this range were not tested; but the tested dose rates correspond to dose ranges used in radiobiology experiments where the total doses are in the range of a few Gy. Personal dose monitors can be used for calibration of low activity sources for very low dose and dose rate exposure. On the basis of a t test, some statistically significant differences were observed between the dose measurements using Gafchromic films and those using the semiconductor dosemeters, for both gamma radiation and X-rays. As a result of measurements performed at different dose rates for X-rays, it can be concluded that calibration of the semiconductor dosemeters is necessary for a given experimental setup.

It should be mentioned that the uncertainties associated with the semiconductor dosemeter measurements are similar to those obtained with the radiochromic films and an ionisation chamber. Although the uncertainty in capacitance is a systematic error that is unique for each individual semiconductor dosemeter board, in the present analyses it was regarded as a random error. The reason for using this approach was that, otherwise, one would have to calibrate the response of each dosemeter, with each capacitor combination, in each radiation field. On the one hand, this would be time-consuming, but, on the other hand, it would increase the accuracy of the measurements and avoid bias between the individual dosemeters. However, it is emphasized again that the scope of this work was not to develop a device allowing for high-precision dosimetry but, on the opposite, to develop a simple dosemeter that allows for measurements at an acceptable level of precision. The semiconductor dosemeters described here allow measurements sufficiently precise in view of the large inherent variability of biological responses to ionizing radiation. If measurements are made with EBT Gafchromic films, their uncertainty ranges from less than 1% to almost 2% (van Battum et al. 2008; Bouchard et al. 2021). These values vary depending on the dose absorbed, the size of an irradiated region, or the quality of the scan performed. The dose measurement procedure using Gafchromic films is a time-consuming process that includes a separate calibration for each of the films used for irradiation and film scanning after 0.5–24 h (Casanova Borca et al. 2013). In contrast, an immediate readout after a dose measurement is possible using an ionization chamber. However, its disadvantage is the need to periodically perform certification processes in accredited testing laboratories. According to manufacturer data (PTW Dosimetry 2019), the long-term stability of an ionization chamber is around 0.5% per year. Undoubtedly, if a dosemeter is intended to provide legally valid dosimetry information, it must undergo regular calibration. Additionally, in certain countries, it may also be required to undergo type testing in accordance with relevant International Electrotechnical Commission (IEC) standards, the necessity of which varies depending on the specific application. Although the precision required in radiobiological experiments is not as high as in clinical practice, for example in measuring the quality of radiotherapy, control dosimetric checks are necessary. Therefore, in-house dosemeters, constructed in an inexpensive way, might be a useful tool for dose validation. The integrating capacitors used in this work to build the dosemeter have an uncertainty of 10% in their capacitance, which translate into a 10% uncertainty in the calculated average response. Using photodiodes of better quality (which are of course more expensive) could reduce the uncertainty of dose measurements down to 2% (Rikner and Grusell 1983; Wilkins et al. 1997) making semiconductor dosemeters more sensitive than ionization chambers (Saini and Zhu 2004). In addition to the dose itself, its uniform delivery to all biological materials is crucial in radiobiological experiments. Therefore, spatial dose homogeneity in irradiation chambers should be also monitored, which can be done with the dosemeter developed in this study.

## Conclusions

With the growing interest in and use of ionizing radiation in medical and nuclear applications, it is important to better understand biological effects of ionizing radiation. In the case of radiobiological experiments, a very important element is determining the value of the dose delivered to any irradiated biological material. This demand is not always met because access to suitable dosemeters is limited. Consequently, there is a need for simple, low-cost dosemeters that most radiation biology laboratories can afford. The presented dosemeter is not meant as a substitute for calibrated, professional dosemeters. Also, when constructed, its precision and reproducibility must be tested. Nevertheless, it was shown here that the instrument can contribute to improved dosimetry in the field of radiation biology through experimental and educational applications.

### Supplementary Information

Below is the link to the electronic supplementary material.

Supplementary file1 (DOCX 2163 KB)

Supplementary file2 (DOCX 61 KB)

## Data Availability

The data supporting the findings of this study are available from the corresponding author, B.B., upon reasonable request.
