# Supplementary material for: Home-made low-cost dosemeter for photon dose measurements in radiobiological experiments and for education in the field of radiation sciences
Source: Radiat Environ Biophys. 2024 Jun 7;63(3):395–404. doi: 10.1007/s00411-024-01076-1 (PMC11341755; doi:10.1007/s00411-024-01076-1)
Supplement: Supplementary file 1 — Supplementary file1 (DOCX 2163 KB) [file 411_2024_1076_MOESM1_ESM.docx]

Electronic Supplement (Annex A)

# A.1. Dosemeter

The dosemeters use silicon diodes for the detection of ionising radiation. When ionizing radiation enters the diode, it may ionize the silicon atoms, releasing electrons. When a voltage is applied to the diodes, free electrons will start to drift across the diodes, giving rise to a current. This current can be stored in different capacitors on the dosemeter board. This allows the dosemeter to store the charge released from the irradiation. When the released charge is in the capacitor, it gives rise to a difference in electric potential. Therefore, by measuring the potential difference over the capacitor, the total released charge can be quantified, which is proportional to the absorbed dose.


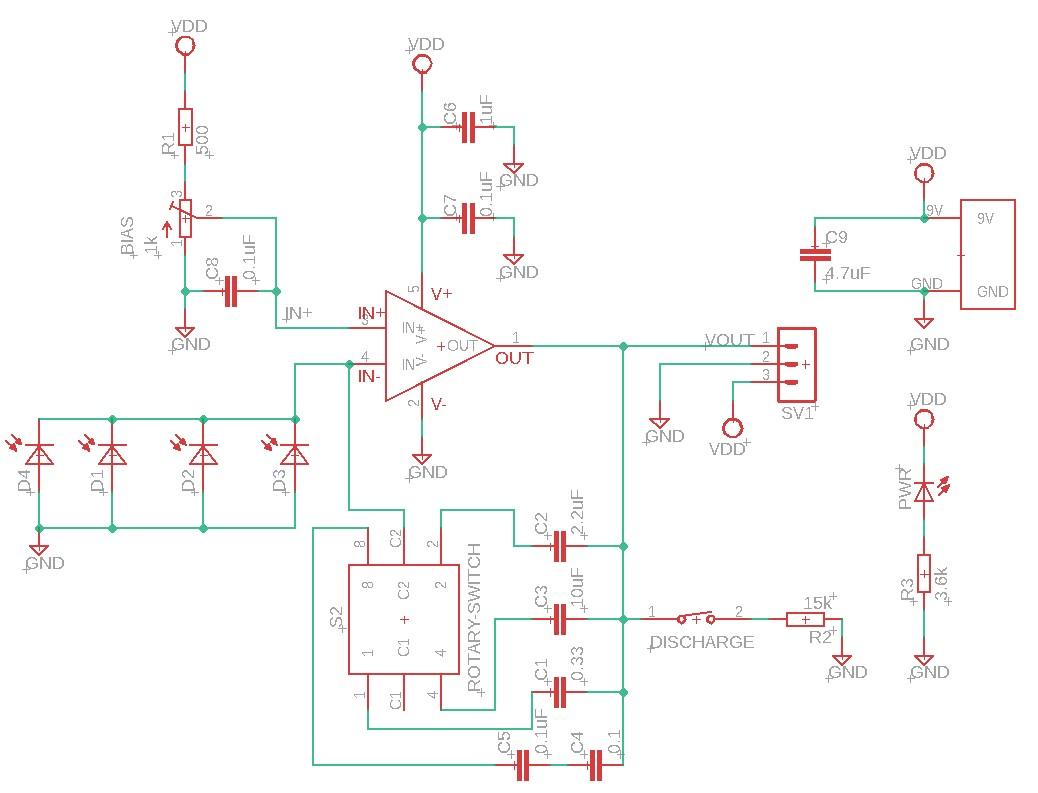


**Fig A1** The schematic of the final dosemeter design.

The dosemeter was built by surface-mounted components on a small printed circuit board (PCB). Since the dosemeters were to be inexpensive, Vishay VBPW34FAS silicon IR photo diodes were chosen, because they had relatively large sensitive area of 7.5 mm^2^ ([www.vishay.com/docs/81127/vbpw34fa.pdf](http://www.vishay.com/docs/81127/vbpw34fa.pdf)). Four diodes were mounted on each board to increase the response compared to the previous design, which used three diodes (Linden, 2018). Footprints for four diodes were introduced on the PCB to allow higher-sensitivity measurements in low-flux conditions. In applications not requiring pinpoint spatial resolution, the PCB may be instead populated with a single diode. For bias voltage supply, a Texas Instruments OPA140AIDBVR single-stage amplifier was chosen, since it had a low input bias current and stable voltage supply (<http://www.ti.com/lit/ds/symlink/opa140.pdf>). The IN+ pin of the amplifier, used to set the bias voltage, was connected to a trimpot in series with a resistor, such that the bias voltage could be adjusted from ground up to 6 V. The schematic of the final dosemeter design is shown in Fig. A1.

For integrating capacitors, tantalum capacitors of different capacitances were chosen. For the previous dosemeter design, both ceramic and tantalum capacitors were used for integrating capacitors. The ceramic capacitors were found to have a non-linear response to the absorbed dose, whereas the tantalum capacitors responded linearly, as can be seen in Fig. A2. (Linden 2018). Hence, tantalum capacitors were chosen. Four capacitors of different capacitances were used to increase the measurable range of dose. To determine what capacitances should be used, a simple equation was derived that allowed to identify the capacitance that would yield a specific voltage for a specific absorbed dose as follows:

$C=\frac{\frac{4}{3}R\left( E \right)C_{0}D}{U}$, (A1)

where $R\left( E \right)$ is the response of the diodes depending on energy, $C_{0}$ is the capacitance used during measurements of $R\left( E \right)$, $D$ is the absorbed dose, $U$ is the voltage over the integrating capacitor and the term 4*/*3 is the expected relative increase of response due to the increase of sensitive area.

Using Eq. A1 suggested that appropriate capacitances would be 50 nF, 0.33 $\mu$F, 2.2 $\mu$F and 10 $\mu$F. These capacitances were chosen such that the doses to be measured would overlap slightly between the capacitors, so that all doses from the smallest to the highest could in theory be measured without any discontinuities. The calculated measurable dose range for each capacitor can be found in Table A1. The lowest and highest dose for each capacitor was set to the dose where 1 V and 8 V had been reached over the capacitor, respectively. It is important to calibrate the dosemeters due to the damages that can be caused by the exposure to ionizing radiation (Goncalves et al. 2020, Jursinic 2019, Jursinic 2023, York et al. 2005).

**Table A1**. Calculated measurable dose range for each capacitor, for photons from an ^241^Am, ^137^Cs, and ^60^Co source. The highest and lowest detectable dose was calculated using 1 V and 8 V, respectively. The dose is given in Gy, and the response was found using $C_{0}$ = 47 pF (Linden 2018).

| **Source** | **^241^Am** | | **^137^Cs** | | **^60^Co** | |
| --- | --- | --- | --- | --- | --- | --- |
| $R\left( E \right)$ **(kV/Gy)** | **312** | | **93** | | **85** | |
| $C$ | ***D_min_*** | ***D_max_*** | ***D_min_*** | ***D_max_*** | ***D_min_*** | ***D_max_*** |
| 50 nF | 0.003 | 0.020 | 0.009 | 0.069 | 0.009 | 0.075 |
| 0.33 $\mu$F | 0.017 | 0.135 | 0.057 | 0.453 | 0.062 | 0.496 |
| 2.2 $\mu$F | 0.113 | 0.900 | 0.378 | 3.020 | 0.413 | 3.304 |
| 10 $\mu$F | 0.512 | 4.092 | 1.716 | 13.73 | 1.877 | 15.02 |


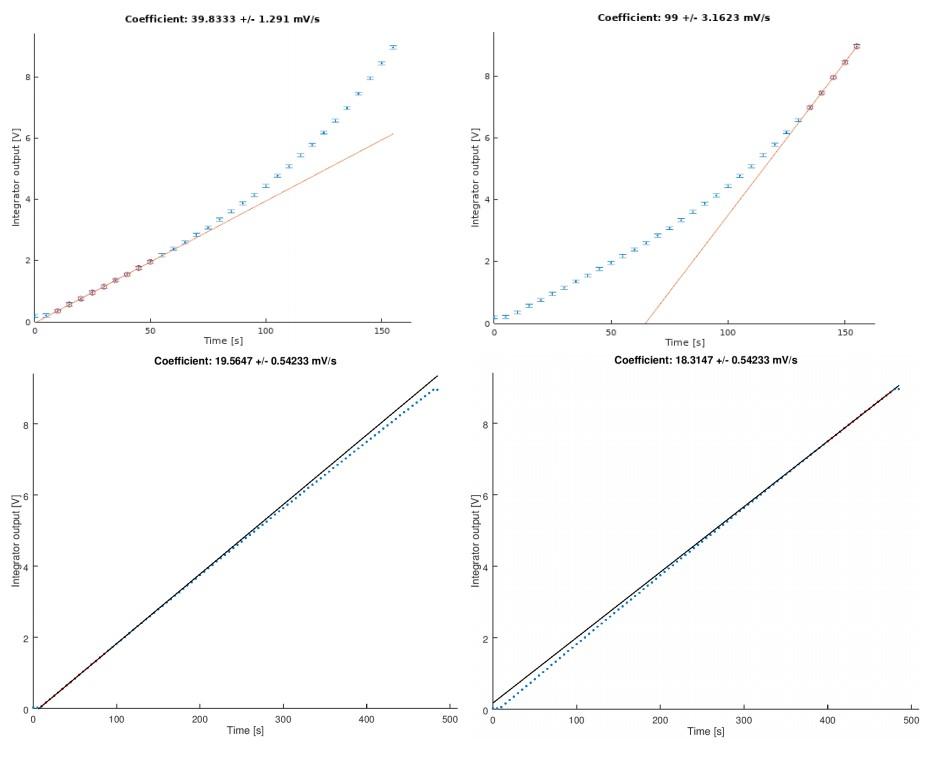


**Fig A2** Comparison of the response for ceramic capacitors (top panels) and tantalum capacitors (bottom panels). The solid lines are linear fits to the dotted data. The response was measured in a constant dose rate field (Linden 2018).

A Multicomp RBM2-16RBVR rotary switch was used to select between the capacitors. The switch was chosen since it had four connections besides the common port, which could be activated in any combination. Thereby, capacitances could be added to further increase the measurable range. A LED was placed on the board to indicate that the board had a working power supply. The LED was connected in series with a resistor to reduce the drawn current. A 3-pin male connector was attached to the board, and was connected to the capacitor voltage net (V_out_), the battery ground (GND) and the battery 9 V port (VDD). This was made in a way that the dosemeter ground could be connected to the dose reader ground, the battery voltage could be checked and the voltage over the capacitors could be measured. On the bottom side of the board the battery connector was mounted. Four decoupling capacitors were mounted on the PCB. The noise on the board was expected to be low, since the board is battery powered and there are only few electronic components, whereof the amplifier is the only one which required power to operate. However, since ceramic capacitors were very cheap, there was no reason not to add them, just in case. Ceramic capacitors were selected for decoupling capacitors since the voltage linearity was not an issue for the purpose they were used.

The decision was made that the dosemeter should have a build-up cap surrounding the diodes to ensure that full dose build-up would be achieved before entering the diode. The cap was made of ABS (acrylonitrile butadiene styrene) plastic, because ABS plastic has a density similar to water: The density of ABS is between 1.02 g/cm^3^ and 1.07 g/cm^3^ (Walker and Burton, 2001), and the density of liquid water at 0 °C is 1.00 g/cm^3^ (Nordling and Carl, 2006). The second reason for using ABS was that there was a 3D printer available that allowed the build-up cap to be printed form-fitted to the dosemeter. The thickness of the cap should be at least equal to the range of the most energetic secondary electrons of the ^137^Cs and ^60^Co radiation fields in order to block these high-energetic electrons, while simultaneously establishing charged-particle equilibrium around the diodes to allow gamma-field calibration in terms of dose-to-ABS (a fair approximation of dose-to-water).

For calculations of the secondary electron- and -particle ranges in the build-up ABS cap, tabulated values of the electron range in polystyrene were used. This was done because tabulated data for polystyrene are widely available, which is not the case for ABS. Also, the elemental compositions of ABS and polystyrene are fairly similar, with ABS containing 40-60% styrene molecules (Walker and Burton, 2001). The highest energy among the photons emitted from the investigated sources is 1.49 MeV (occurring during the decay of ^60^Co) (ICRP, 1983). Using tabulated values of the continuous slowing down approximation (CSDA) range for electrons in polystyrene (ICRU, 1984), the CSDA range was found to be 4.9 mm. Therefore, the build-up cap was made 5 mm thick. The final design can be seen in Fig. A3. A photo of the dosimeter is shown in Fig. A4.


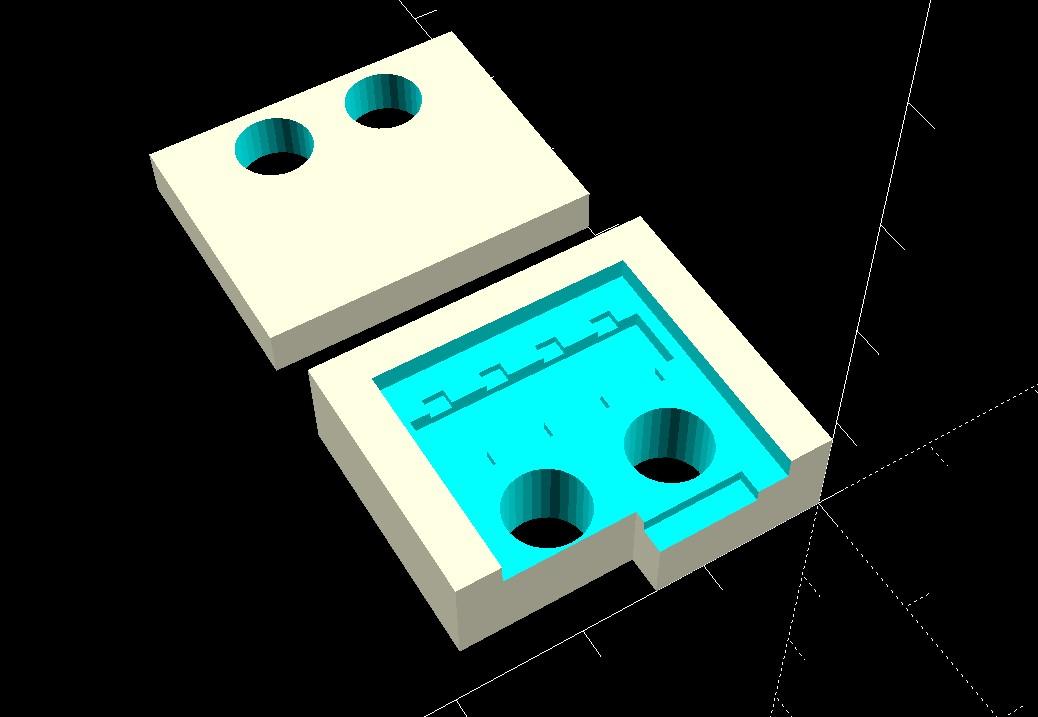


**Fig A3** Design of the ABS (acrylonitrile butadiene styrene) build-up cap. Holes in the top piece are for the row of diodes and their solder joints.


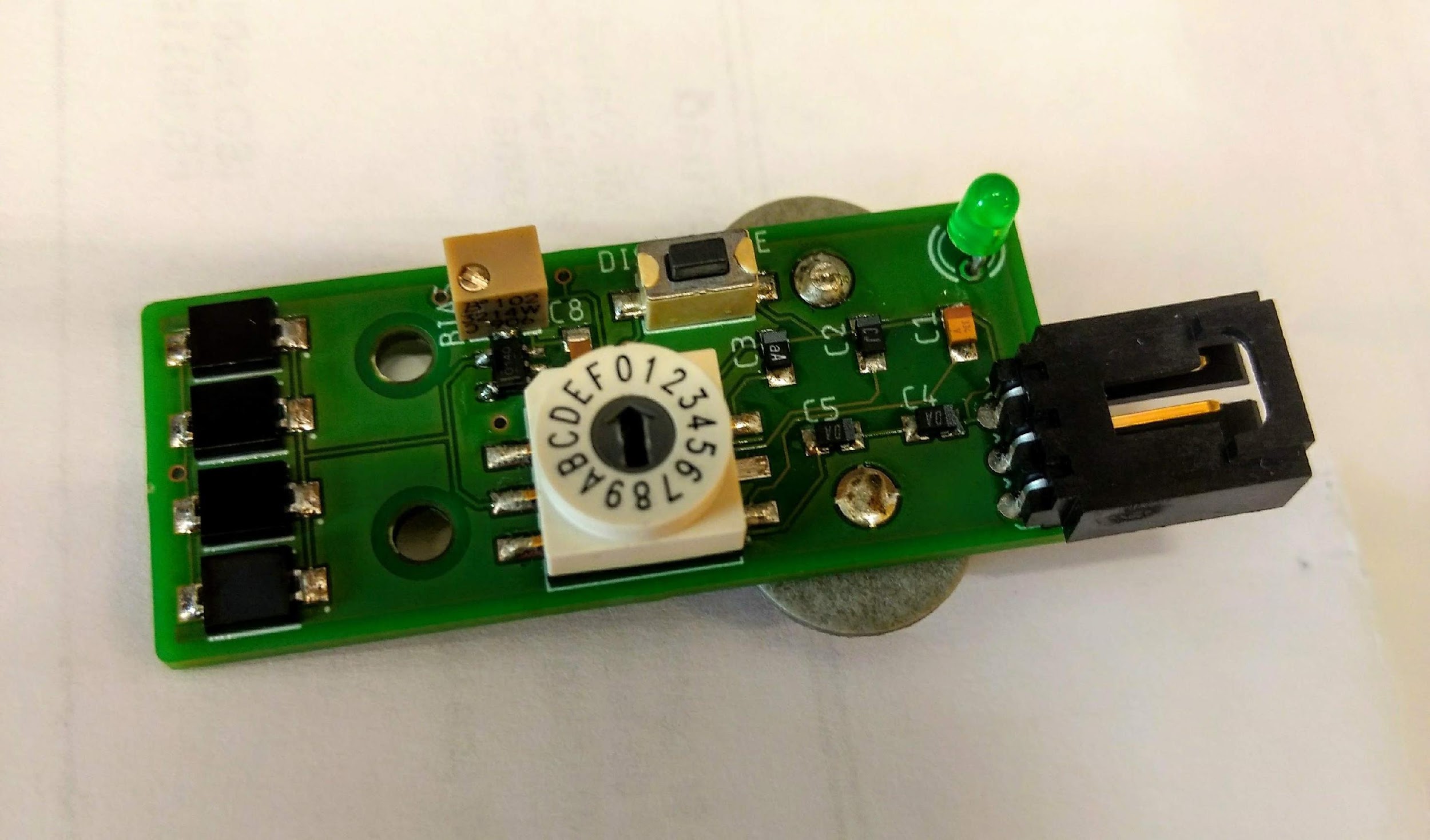


**Fig A4** Fully assembled dosemeter without build-up cap. A picture with the build-up cap mounted can be found in Fig. 1 of the main manuscript. To the left are the photo diodes. Just above the rotary switch to the left is the amplifier and the trimpot used to supply bias voltage. The discharge button is just to the right of the trimpot. The integrating capacitors are located to the right of the rotary switch. Above the integrating capacitors is a button used to discharge the capacitors before each measurement, and to the right is an LED that indicates whether or not the dosemeter has power. The dimensions of the board are 20 mm x 48 mm.

# A.2. Dose reader

The dose reader used is based on an Arduino Uno microcontroller board (Fig. A5). The Arduino was chosen since it is inexpensive, widely available, includes plenty of after-market plug-in components, and uses an open-source programming software. Also, the Arduino can read analog signals up to 5 V with a resolution of about 5 mV ([www.arduino.cc/reference/en/language/functions/analog-io/analogread/](http://www.arduino.cc/reference/en/language/functions/analog-io/analogread/)). An Adafruit LCD module with five programmable buttons was added to the Arduino (<https://cdn-learn.adafruit.com/downloads/pdf/rgb-lcd-shield.pdf>). Cables were soldered to the GND, A0 and A1 pins of the Arduino. GND was to be connected to the dosemeter ground. A0 and A1 are analog input pins that were used to read the capacitor voltage (V_out_) and the dosemeter battery voltage (VDD), respectively. A voltage divider was built on a small bread board together with the pin contact. It was done since the Arduino could only read voltages of at most 5 V, and the expected voltage on the dosemeter was expected to reach 9 V. Using two 1 k$\Omega$ resistors in series for each input, the voltage divider was expected to reduce the voltage on the dosemeter to half the dosemeter voltage before being read by the Arduino.


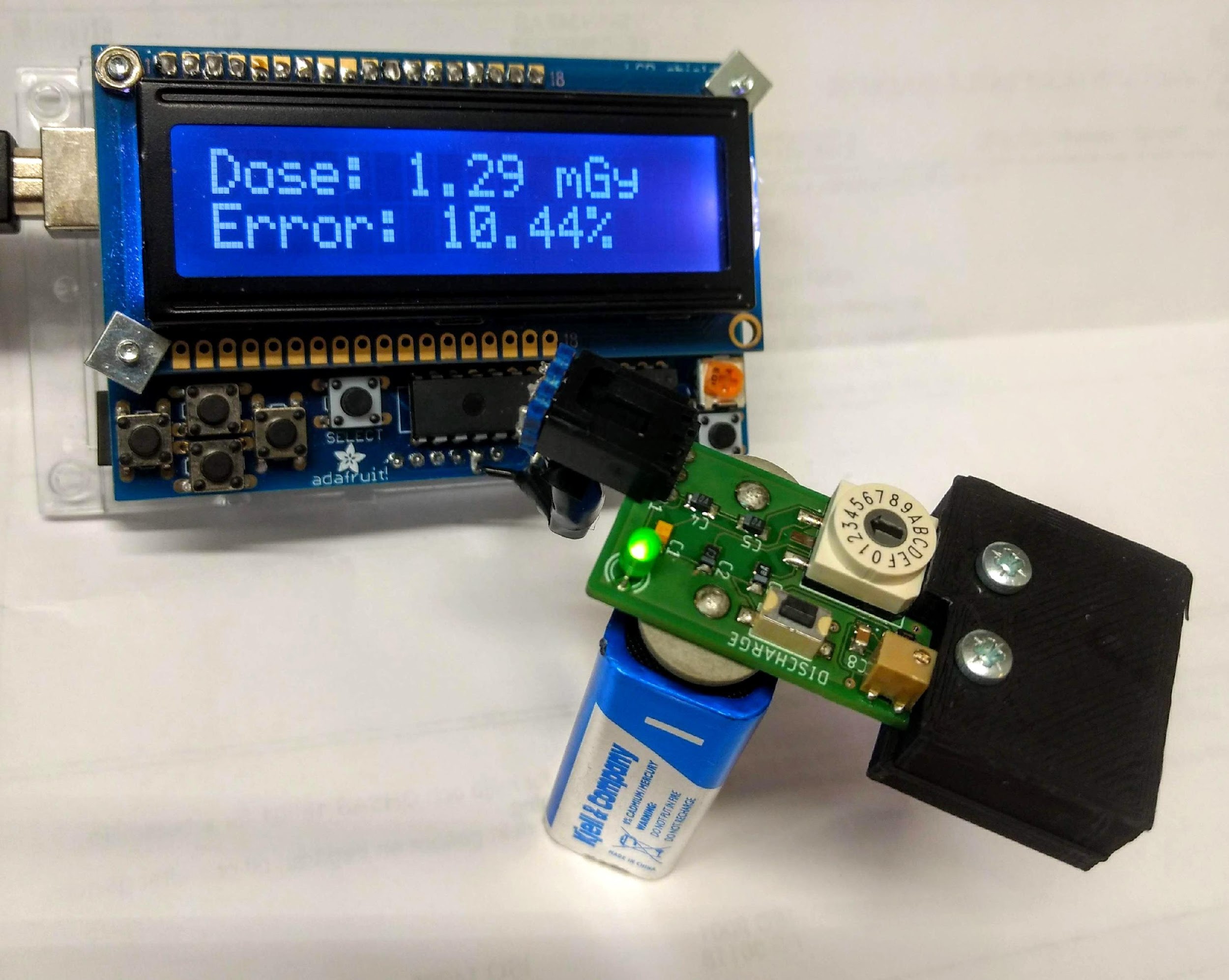


**Fig A5** Dose reader connected to the dosemeter.

There are multiple ways to power an Arduino. Throughout the project, the Arduino was powered by connecting it to the USB outlet on a laptop, since this allowed the measured data to be saved to the computer. For future use, it was decided that the Arduino should be battery powered for convenience. According the specifications of the Arduino, the battery voltage should be kept between 7 V and 12 V to protect the Arduino from over voltage damage, but ensuring that the 5 V reference voltage stays stable (<https://store.arduino.cc/arduino-uno-rev3>). Since the dosemeters required use of 9 V batteries, it was decided to use the same batteries for the Arduino. Consequently, a connection cable was soldered to the second GND pin and the V_in_ pin. The V_in_ pin was then connected to the A2 analog input pin via a voltage divider so that the supply voltage could be measured (Fig. A6).

The dose reader was programmed such that when powered, it would immediately measure the voltage on the input pins. Every time a voltage is to be measured, the voltage on the input pin is measured 30 times in short succession. The average value and its standard deviation is then calculated and stored. This was implemented to reduce the uncertainty of the measured voltage due to noise.

First, the supply voltage of the Arduino is evaluated. If the voltage was found to be lower than 7 V, the dose reader would display a warning, recommending the user to change the battery. This was implemented since at supply voltages lower than 7 V, it could not be guaranteed that the reference voltage on the Arduino is still close to the intended 5 V. Next, VDD was evaluated. Since the response of the dosemeter depends on the bias voltage, which in turn is linearly dependent on VDD, a warning is displayed if VDD is too low, to ensure a stable response. Finally, V_out_ is evaluated, and if it is too close to the VDD value, the dose reader would display a warning that the capacitor may be saturated, making the measurement unreliable.

Since the voltage increase during irradiation is dependent on the photon energy and capacitance used, a menu system was implemented that requires the user to select the used source and the capacitance used during irradiation. The source menu includes some pre-defined sources, ^137^Cs, ^60^Co and X-ray (being the X-ray machine used during calibration), and an option to manually input the used photon energy. If the photon energy is manually entered, the dose is calculated using a function fitted to the measured response as a function of photon energy. When the source has been selected, the capacitance is requested. The menu contains all the dial positions printed on top of the rotary switch. When both source and capacitance have been entered, the absorbed dose with its uncertainty are calculated using the measured average voltage over the capacitors according to Equations 1 and 2 in the main manuscript.

The results are displayed and switched between displaying the calculated dose and the measured voltages every five seconds. Note that this program was not used during the testing of the dosemeter: During testing the voltage was measured but not converted to dose.


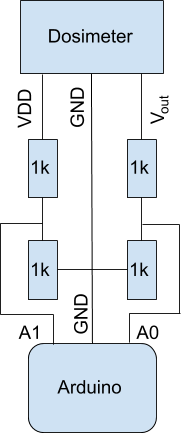


**Fig. A6** Simplified schematic of the voltage divider used to bring down the voltage on the dosemeter board to the measurable range of the Arduino.

**A.3. Using the dosemeter**

Before performing measurements with the dosemeter, one must ensure that the capacitors are empty. This is done by pressing the discharge button for a few seconds when the battery is disconnected. This must be done before each irradiation!

Depending on the radiation dose to be measured, an appropriate capacitance must be selected to set the desired range. Approximate ranges provided by each individual capacitor for different radiation sources and doses can be found in Table A2, while Table A3 includes some common rotary switch settings and their corresponding capacitance. When the capacitance is selected, the battery must be connected and the dosemeter must be placed in the radiation field. This should be done in a way that the diodes are normal to the radiation field as in Fig. A7, since an incident angle less than 90° may affect the effective detector area relative to the photon source.

The dosemeter should preferably be placed at the reference position of the irradiation chamber (usually the centre of the chamber), but this is not strictly necessary, as long as the diodes are normal to the field.

**Table A2** Calculated measurable dose range in different fields, using the measured response to ionizing radiation in each radiation field. The dose is given in units of Gy.

| **Capacitor** | ‍**Value** ($\mu$F) | **80 keV X-rays** | **^137^Cs** | **^60^Co** |
| --- | --- | --- | --- | --- |
| C1 | 0.33 | 0.01 - 0.3 | 0.03 - 0.7 | 0.025 - 0.63 |
| C2 | 2.2‍ | 0.08 - 2.0 | 0.18 - 4.61 | 0.163 - 4.17 |
| C3 | ‍10 | 0.36 - 9.2 | 0.82 - 20.9 | 0.74 - 19 |
| C4 | 0.05‍ | 0.002 - 0.046 | 0.004 - 0.10 | 0.004 - 0.01 |

**Table A3** Different dial positions in which capacitors are used and total capacitance.

| **Dial** | **Capacitor** | **Capacitance (**$\mu$**F)** |
| --- | --- | --- |
| 0 | None | 0 |
| 1 | C2 | 0.33 |
| 2 | C1 | 2.53 |
| 3 | C1+C2 | 0.05 |
| 4 | C3 | 10 |
| 8 | C4 | 0.05 |


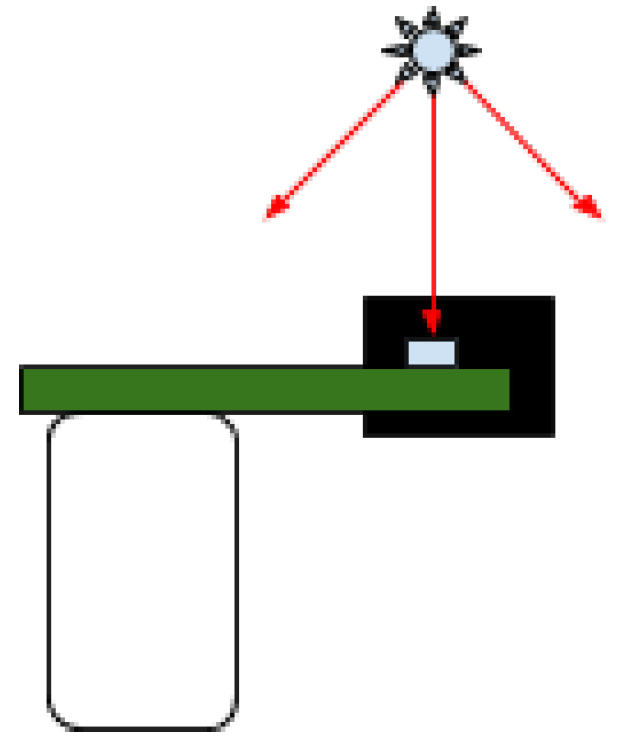


Fig A7 Illustration of the ideal case where the radiation field is normal to the diode surface. The plastic cap attached to the dosemeters is shown in black. The white rectangle in the cap illustrates the location of the silicon diodes.

When the dose reader is powered on, the user will have to press the "select" button to perform a measurement of the voltage on the dosemeter board. For this the dosemeter must be connected according to Fig. A8 (note the color of the cables).


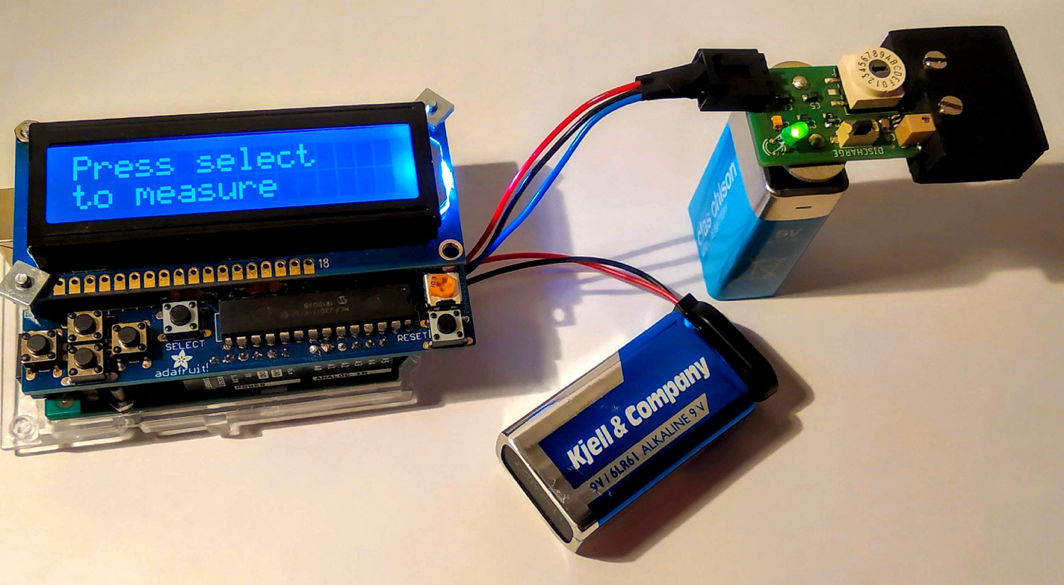


**Fig A8** Properly connected measurement system.

The dose reader may display two different warnings. One possibility is that the capacitor is saturated. This means that the capacitor is full of charge and cannot store any more electrons. This is problematic, as the user cannot know when the dosemeter reached saturation. If it is in the middle of the irradiation time, then the assured dose will be lower than the true dose. In such a case it is recommended to repeat the measurement with increased capacitance.

Another possible warning is that the battery voltage of the dosemeter is low. This is problematic because in such a case the response of the diodes to ionising radiation may decrease and, consequently, the dose may again be underestimated. Note that the battery voltage will be displayed by the dose reader after a measurement. Therefore, it is recommended to check the battery status regularly and replace the battery if it is significantly lower than the recommended lowest voltage of 7.5 V.

The user is then prompted to input the radiation source used during irradiation. This allows the dose reader to select the appropriate response value to be uses during dose calculations. Currently, the dosemeter is only calibrated in fields from three radiation sources: ^137^Cs, ^60^Co, and an 80 keV X-ray tube belonging to the Department of Molecular Biosciences at Stockholm University.

After selecting the radiation source, the user must input the position of the rotary switch during irradiation, which will inform the dose reader which capacitance is used. Finally, the calculated dose is displayed together with its calculated uncertainty. The voltage over the capacitors is then shown as "Vout", and the battery voltage as "Batt". The display will change between showing the dose and the voltages every 5 seconds. The voltages are mainly of interest if the dose reader has warned the user about low battery voltage. It can also be used to check if the devices are connected in the correct way (Vout should never be greater than Batt).

To perform a new measurement, the reset button in the lower right corner must be pressed.

**References**

Goncalves JAC, Mangiarotti A, Bueno CC (2020) Current response stability of a commercial PIN photodiode for low dose radiation processing applications. Radiat. Phys. Chem. 167, 108276–108279

Jursinic P (2019) PIN diodes for radiation therapy use: Their construction, characterization, and implementation. Phys Med. Mar;59:86-91. doi: 10.1016/j.ejmp.2019.02.021.

Jursinic P. (2023) A PIN photodiode ionizing radiation detector with small angular dependence and low buildup. Rad. Meas. 166:1-6

Linden F (2018) Construction and characterization of radiation detector systems, M.Sc thesis, Department of Physics, Stockholm University

Nordling JÖ, Carl J (2006) Physics handbook for science and engineering, 8 red. Stockholm: Studentlitteratur AB.

Radiation dosimetry: Electron beams with energies between 1 and 50 Mev, ICRU, 7910 Woodmont Ave. (1984). International Commission on Radiation Units and Measurements, 35.

Task Group on Dose Calculations, International Commission on Radiological Protection (ICRP). (1983). Radionuclide Transformations: Energy and Intensity of Emissions, 11.

Walker B, Burton L (2001). Polyvinyl acetate, alcohol, and derivatives, polystyrene, and acrylics. Patty’s Toxicology.

York E, Alecu R, Ding L, Fontenla D, Kalend A, Kaurin D, et al. (2005) AAPM Report No. 87 Diode in Vivo Dosimetry for Patients Receiving External Beam Radiation Therapy. (2005). American Association of Physicists in Medicine, College Park, MD.
