# Supplementary material for: Home-made low-cost dosemeter for photon dose measurements in radiobiological experiments and for education in the field of radiation sciences
Source: Radiat Environ Biophys. 2024 Jun 7;63(3):395–404. doi: 10.1007/s00411-024-01076-1 (PMC11341755; doi:10.1007/s00411-024-01076-1)
Supplement: Supplementary file 2 — Supplementary file2 (DOCX 61 KB) [file 411_2024_1076_MOESM2_ESM.docx]

Electronic Supplement (Annex B)

To confirm the suitability of Gafchromic films to measure gamma radiation of different energy and dose rates, the data obtained for the sources used in this work are compared with the data provided in (Rink et al., 2007). The optical density values determined for films irradiated with a dose of 1 Gy using various photon sources are presented in Fig. B1. Given the involved experimental uncertainties, there is reasonable agreement between the results of the measurements, which means that the response of the radiochromic films is independent of the energy or dose rate.


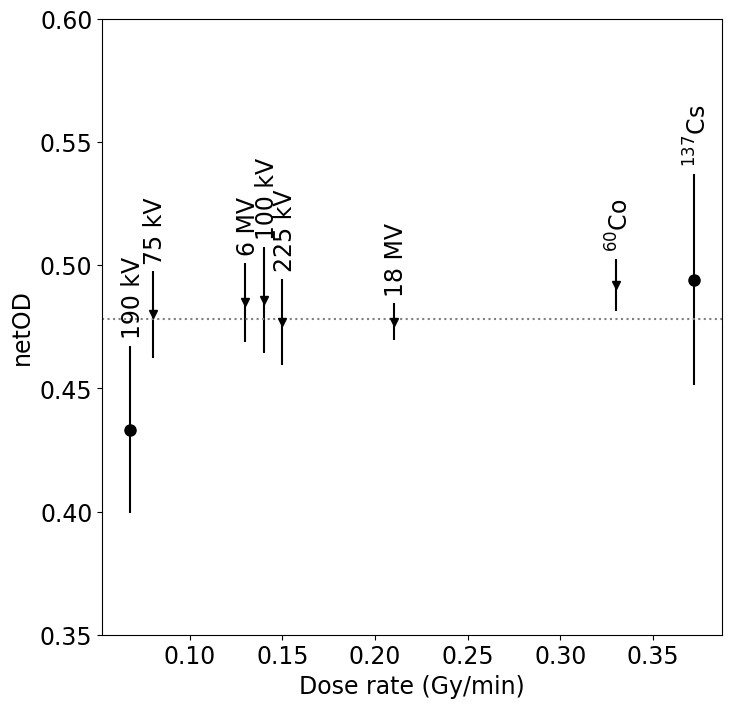


**Fig B1** Optical density as a function of dose rates obtained at a dose of 1 Gy using different radiation sources. The circle points indicate measurements with the X-ray tube (190 kV) and the Cs-137 source used in this work. The error bars represent uncertainties calculated on the basis of the error propagation method.

**References**

Rink A, Vitkin A, Jaffray DA (2007) Energy dependence (75 kVp to 18 MV) of radiochromic films assessed using a real-time optical dosimeter. Med Phys. 34(2), 458-63. <https://doi.org/10.1118/1.2431425>
